# Evaluation of the Biological Activities of Peptides from Epidermal Mucus of Marine Fish Species from Chilean Aquaculture

**DOI:** 10.3390/md22060248

**Published:** 2024-05-28

**Authors:** Claudio A. Álvarez, Teresa Toro-Araneda, Juan Pablo Cumillaf, Belinda Vega, María José Tapia, Tanya Roman, Constanza Cárdenas, Valentina Córdova-Alarcón, Carlos Jara-Gutiérrez, Paula A. Santana, Fanny Guzmán

**Affiliations:** 1 Laboratorio de Cultivo de Peces Marinos, Facultad de Ciencias del Mar, Universidad Católica del Norte, Coquimbo 1781421, Chile; claudio.alvarez@ucn.cl; 2 Laboratorio de Fisiología y Genética Marina (FIGEMA), Centro de Estudios Avanzados en Zonas Áridas (CEAZA), Coquimbo 1781421, Chile; teresa.toro@alumnos.ucn.cl (T.T.-A.); belinda.vega@ceaza.cl (B.V.); maria.tapia01@alumnos.ucn.cl (M.J.T.); valentina.cordova@ug.uchile.cl (V.C.-A.); 3 CRC Innovación, Puerto Montt 5507642, Chile; jpnenen@gmail.com; 4 Núcleo Biotecnología Curauma, Pontificia Universidad Católica de Valparaíso, Valparaíso 2373223, Chile; tanya.roman.b@mail.pucv.cl (T.R.); constanza.cardenas@pucv.cl (C.C.); 5 Genomics on the Wave SpA, Viña del Mar 2520056, Chile; 6 Centro Interdisciplinario de Investigación Biomédica e Ingeniería para la Salud—MEDING, Universidad de Valparaíso, Valparaíso 2362905, Chile; carlos.jara@uv.cl; 7 Facultad de Medicina, Escuela de Kinesiología, Universidad de Valparaíso, Valparaíso 2362905, Chile; 8 Instituto de Ciencias Aplicadas, Facultad de Ingeniería, Universidad Autónoma de Chile, Santiago 8910060, Chile

**Keywords:** mucus, peptide, *Seriola lalandi*, *Seriolella violacea*, antimicrobial, antioxidant, respiratory burst

## Abstract

The skin of fish is a physicochemical barrier that is characterized by being formed by cells that secrete molecules responsible for the first defense against pathogenic organisms. In this study, the biological activity of peptides from mucus of *Seriola lalandi* and *Seriolella violacea* were identified and characterized. To this purpose, peptide extraction was carried out from epidermal mucus samples of juveniles of both species, using chromatographic strategies for purification. Then, the peptide extracts were characterized to obtain the amino acid sequence by mass spectrometry. Using bioinformatics tools for predicting antimicrobial and antioxidant activity, 12 peptides were selected that were chemically produced by simultaneous synthesis using the Fmoc-Tbu strategy. The results revealed that the synthetic peptides presented a random coil or extended secondary structure. The analysis of antimicrobial activity allowed it to be discriminated that four peptides, named by their synthesis code 5065, 5069, 5070, and 5076, had the ability to inhibit the growth of *Vibrio anguillarum* and affected the copepodite stage of *C. rogercresseyi*. On the other hand, peptides 5066, 5067, 5070, and 5077 had the highest antioxidant capacity. Finally, peptides 5067, 5069, 5070, and 5076 were the most effective for inducing respiratory burst in fish leukocytes. The analysis of association between composition and biological function revealed that the antimicrobial activity depended on the presence of basic and aromatic amino acids, while the presence of cysteine residues increased the antioxidant activity of the peptides. Additionally, it was observed that those peptides that presented the highest antimicrobial capacity were those that also stimulated respiratory burst in leukocytes. This is the first work that demonstrates the presence of functional peptides in the epidermal mucus of Chilean marine fish, which provide different biological properties when the fish face opportunistic pathogens.

## 1. Introduction

In Chile, spearheading initiatives, such as the Chilean Aquaculture Diversification Programs (PDACH), have actively fostered the commercial farming of native species such as *Seriola lalandi* (yellowtail kingfish) and *Seriolella violacea* (palm ruff) because of their fast growth, adaptability to various growing conditions, and good commercial value [1,2,3,4]. Indeed, it becomes imperative to proactively address health concerns as these species undergo intensive cultivation and aquaculture practices intensify the inherent risks associated with disease proliferation.

While wild fish commonly bear a burden of parasites, the controlled environment of aquaculture tends to exacerbate this exponentially. This heightened parasite presence adversely affects the immune system, predisposing the fish to secondary infections caused by bacteria, viruses, and fungi [5]. Specifically focusing on *S. lalandi*, parasites such as *Caligus lalandei* and *Zeuxapta seriousolae* have been identified, residing in the gills and skin, respectively [6]. Moreover, the intensification of aquaculture practices creates a conducive environment for the proliferation of bacteria belonging to the genus Vibrio. These microorganisms, known for their ability to independently grow and reproduce in water, pose a significant threat to the health of cultivated species [7,8]. Indeed, in the cultivation of yellowtail kingfish in China, the emergence of shoots affected by vibriosis-related diseases has been documented [6].

The fish tegument, comprising layers like the cuticle, epidermis, basal membrane, dermis, and hypodermis, acts as a defensive barrier [9,10]. The cuticle, derived from secretions of epithelial and calciform cells, contains mucopolysaccharides, specific immunoglobulins, and fatty acids [11]. The epidermis, a non-keratinized stratified flat epithelium, is primarily cellular in structure [10]. Playing a crucial role in superficial wound repair, the skin and mucus collaborate in the healing process. Immediately following an injury, mucus laden with numerous lymphocytes covers the wound [9]. Subsequently, marginal wound cells proliferate, forming a protective layer that gradually completes the healing process [9]. Mucus secretions, encompassing proteins like mucin glycoproteins, agglutinins, reactive C protein, immunoglobulins (IgM), and enzymes, such as peroxidase, serve as physical protectors and immunological effectors [12]. Additionally, the mucus contains energetic molecules providing information on glucose metabolism and the internal metabolic state of the animals. It also contains molecules related to protein metabolism and defense against infections, including antimicrobial peptides [13]. Studies on marine species like juvenile gilthead sea bream, European sea bass, and meagre have demonstrated the antimicrobial activity of mucus [13]. However, a more in-depth knowledge of the specific molecules within epidermal mucus is essential to identify their role as a defense mechanism against pathogens and to assess the overall health of the fish. Peptides constitute a significant category of bioactive molecules [14].

Among the extensively studied peptides in diverse animal species are antimicrobial peptides (AMPs), also recognized as host defense peptides (HDPs) [15,16]. Characterized by their small size (less than 10 kDa), AMPs can either be encoded in the genome or generated through the proteolysis of larger polypeptides [17]. They are highly conserved and are produced across a spectrum of organisms, ranging from higher vertebrates to plants [16], with their expression identified in tissues of organs such as the kidney, skin, gills, and intestine. The amino acid sequence of each peptide imparts distinct biological properties, and some peptides exhibit multifunctionality [18]. HDPs may possess antioxidant, antihypertensive, immunomodulatory, antimicrobial, antifungal, and anticoagulant properties [18]. In teleost fish, 19 peptide families with antimicrobial, immunomodulatory, and/or antioxidant activities have been identified. These peptides demonstrate diverse secondary structures, contributing to their multifunctionality and versatility in biological activities [16].

This work focuses on the identification and characterization of peptides derived from the epidermal mucous secretions of aquaculture species, specifically *S. Lalandi* and *S. violacea*. After successful identification, these peptides were chemically synthesized and their antimicrobial and antioxidant properties and the ability to regulate respiratory burst in fish leukocytes were systematically assessed. The main objective of this comprehensive approach is to deepen the understanding of protective capacities inherent in these peptides sourced from fish mucus, with a vision towards their potential utilization as novel marine drugs, leveraging their multifunctional properties.

## 2. Results

### 2.1. Identification and Characterization of Peptides in Epidermal Mucus of S. lalandi and S. violacea

The peptidomic analysis identified a total of 237 peptides in the mucus of *S. violacea* and 52 peptides for *S. lalandi*. The obtained peptide sequences were input into online databases equipped with algorithms that evaluate net charge, amphipathicity, presence of basic amino acids like lysine and arginine, and the presence of amino acids such as tryptophan and cysteine. Following this theoretical comprehensive analysis, 12 peptides were selected to investigate in vitro activity, as detailed in Table 1.

The selected peptides underwent chemical synthesis to validate their biological activity. Each peptide was assigned a synthesis code, as outlined in Table 1. To ensure correct synthesis and purity, mass chromatograms and reverse-phase HPLC spectra were recorded (Supplementary Figures S1 and S2). During the analysis, the retention time (RT) was compared, revealing that peptides with numbers 5069, 5070, and 5078 exhibited the highest hydrophobicity with an RT of 5.8 min. Conversely, peptides 5066 and 5080, with retention times of 4.0 and 4.3 min, respectively, were identified as the most hydrophilic (Table 1).

**Figure 1:**
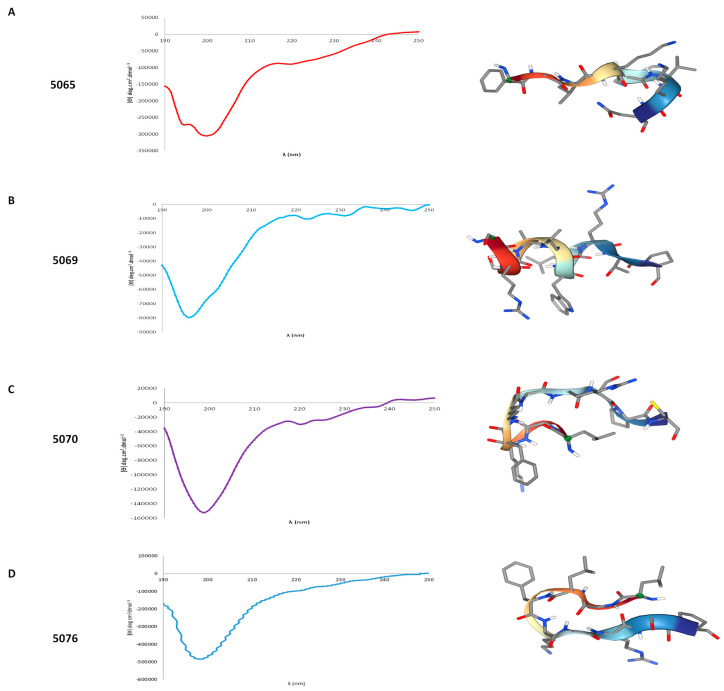
Characterization of secondary structure of peptides. Spectra of circular dichroism for peptides (**A**) 5065; (**B**) 5069; (**C**) 5070; and (**D**) 5076 in 30% *v*/*v* trifluoroethanol in water (left). On the right, 3D-structure model of peptides.

In silico modeling indicated that none of the peptides exhibited a defined secondary structure, implying the absence of α-helix or β-sheets (Figure 1 and Supplementary Figure S3). This finding was corroborated by CD analysis for four of the peptides (Figure 1), which showed characteristic spectra with a minimum between 190 and 200 nm, indicative of peptides with a random coil structure.

### 2.2. Antimicrobial Activity of Peptides Identified in the Mucus of S. lalandi and S. violacea

**Figure 2:**
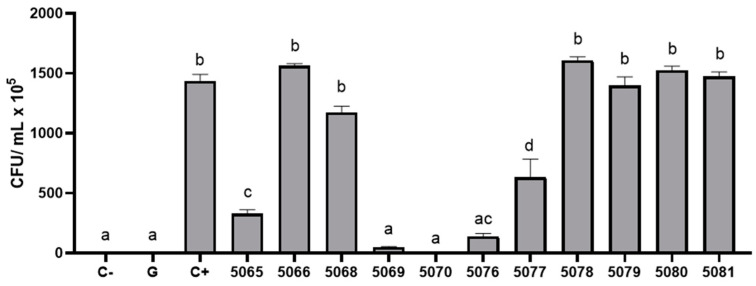
Antibacterial activity of synthetic peptides identified in the mucus of *S. lalandi* and *S. violacea* against *Escherichia coli*. The growth of *E. coli* is expressed in colony-forming units (CFU/mL) after application of 100 µM of synthetic peptides. As a negative control (C−) culture media without bacterial inoculum was used. Positive control (C+) was growth of E. coli without treatment. Treatment with gentamicin was used as positive antibacterial activity (G). Significant differences with respect to C+ are indicated with a different letter (*p* > 0.05).

To conduct an initial screening of the antibacterial activity of the 12 synthesized peptides, their capacity to inhibit the growth of *Escherichia coli* at a concentration of 100 µM was assessed. The results indicated that peptides 5069, 5076, 5065, and 5070 were the greatest inhibitors of *E. coli* growth (Figure 2). Conversely, the remaining peptides exhibited similar or higher bacterial growth compared to the untreated control.

For the four peptides that showed activity, MIC was determined for three bacterial strains (Table 2). Peptide 5065 exhibited an MIC of 6.25 µM against *V. ordalii*, 12.5 µM against *V. anguillarum*, and 25 µM against *E. coli.* Peptide 5069 had the same MIC of 12.5 µM for the two Vibrio strains and 25 µM against *E. coli*. Finally, peptides 5070 and 5076 shared the same MIC of 25 µM against all bacteria, with the exception that peptide 5076 exhibited no activity against *V. anguillarum* at the maximum peptide concentration tested here (100 µM).

**Figure 3:**
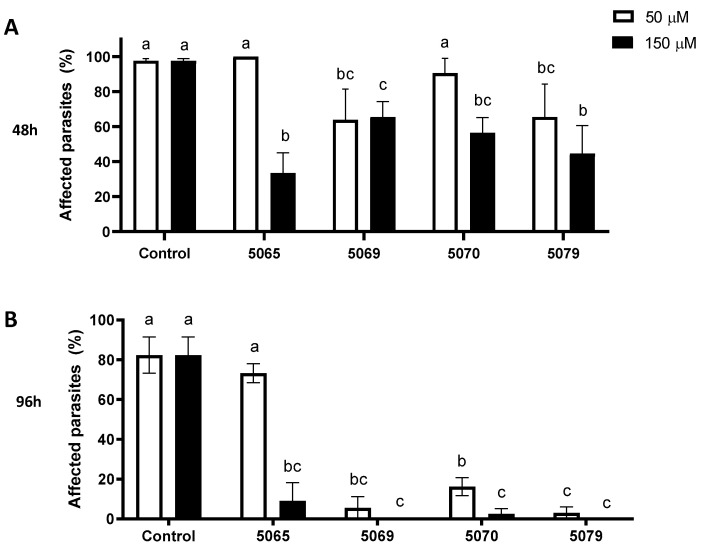
Antiparasitic activity of synthetic peptides against copepodite stage of *C. rogercresseyi*. Affected parasites was expressed as a percentage. The effect of the peptides at 50 and 150 µM on the copepodite stage was evaluated at (**A**) 48 h and (**B**) 96 h. Seawater-only conditions were employed as the control for copepodite survival. Statistically significant differences from the control are indicated by distinct letters (*p* > 0.05).

For the peptides exhibiting antibacterial activity, their antiparasitic efficacy against the ectoparasite *C. rogercresseyi* was investigated. The activity was assessed after 48 h (Figure 3A) and 96 h (Figure 3B) of exposing *C. rogercresseyi* copepods to the synthetic peptides at concentrations of 50 µM and 150 µM. The results showed that, after 48 h, peptides 5069 and 5079 successfully reduced the survival percentage of *C. rogercresseyi* copepods but only at a concentration of 150 µM. Meanwhile, at 96 h post-exposure, peptides 5069, 5070, and 5079 exhibited antiparasitic activity at both concentrations evaluated. Notably, peptides 5069 and 5079 achieved almost 100% effectiveness at 50 µM. It was also observed that peptide 5065 only exhibited activity at 150 µM (Figure 3).

### 2.3. Antioxidant Activity of Peptides Identified in the Mucus of S. lalandi and S. violacea

Often, the term “antimicrobial” falls short in describing the diverse range of properties exhibited by peptides derived from natural sources, as novel functionalities continue to be unveiled. Hence, in our research, we extend our investigation to encompass the assessment of the antioxidant capabilities of these molecules as well. The antioxidant activity of the synthetic peptides was assessed using the DPPH and TRAP methods, with a comparison to the antioxidant capacity of a control peptide 4340 of marine origin *[19]* at the same concentration. The results from both assays are summarized in Table 3.

For the DPPH method, peptides 5065 and 5081 exhibited similar activity to the control peptide. Conversely, peptides 5066, 5067, 5070, and 5077 demonstrated the highest antioxidant activity (*p* > 0.001). Among these four peptides, 5067 was the most effective, showing five times higher antioxidant activity than the control peptide, with a percentage radical scavenging value (%RSC) of 31.03%, compared to 5.48% for 4340 (*p* > 0.001). The remaining peptides did not display significant activity.

In the TRAP method, once again, peptides 5066, 5067, 5070, and 5077 showcased the highest antioxidant activity, with 5067 and 5077 exhibiting 10 times higher antioxidant activity than the control peptide 4340 (*p* > 0.001). The other peptides evaluated demonstrated very low activity, and even the activity of peptide 5068 was below the detection limit of the test.

### 2.4. The Respiratory Burst Stimulation Capacity of Leukocytes by Peptides from S. lalandi and S. violacea

The peptides demonstrating the highest antimicrobial and antioxidant activity were chosen to investigate their potential modulation of respiratory burst in fish leukocytes. Primary cultures of the anterior kidney were employed for this purpose.

**Figure 4:**
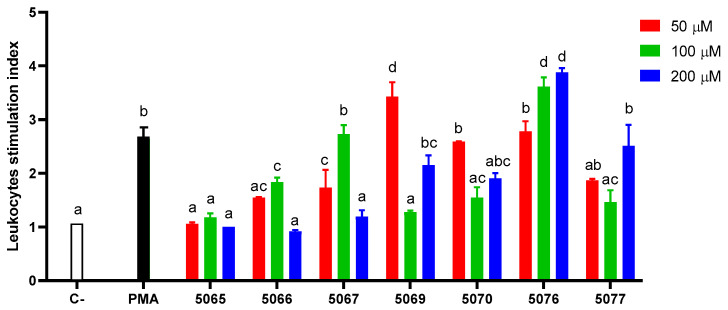
Effect of synthetic peptides on the activation of the respiratory burst process of anterior kidney leukocytes. Respiratory burst stimulation index of leukocytes treated with phosphate buffered saline (C−), Phorbol 12-myristate 13-acetate (PMA), and synthetic peptides (5065–5077) at three different concentrations (50, 100, and 200 µM) at 22 °C. Significant differences are indicated in different letters (*p* > 0.05).

The results are presented in terms of the stimulation index, representing the fold change compared to the unstimulated control. Peptide treatments were compared against the positive control (PMA-stimulated cells). The results indicate that peptides 5067 at a concentration of 100 µM, 5070 at 50 µM, and 5076 at 50 µM exhibited a respiratory burst stimulation similar to the positive control (*p* > 0.05). Additionally, peptides 5069 at 50 µM and 5076 at 100 and 200 µM demonstrated the most substantial stimulation of respiratory burst (*p* > 0.05) (Figure 4).

## 3. Discussion

AMPs play a crucial role as innate immune system mediators in fish, particularly in protecting the mucosa of teleost *[16]*. This research conducted on the epidermal mucus of the marine fish species *Seriola lalandi* and *Seriolella violacea* identified and synthesized 12 peptides that were subsequently subjected to functional properties assessment. 

Because fish mucus may contain compounds originating from the coexisting microbial community, directly attributing the identified sequences to the hosts is challenging. Regarding the observed homology in the blast search, it is crucial to consider that the sequences are relatively short, making matches easily identifiable. Very few peptides exhibit 100% identity in blast searches. Furthermore, when the blast excludes bacterial taxa, similar levels of homology are observed with other organisms such as plants, insects, fungi, parasites, and crustaceans. Similarly, restricting the search to teleost taxa (teleost fishes (taxid: 32443)) yields comparable results, with identity percentages falling within the same range as the previous searches. To conclusively determine whether the peptides correspond to the specific species we studied, data from the genomes, transcriptomes, or proteomes of these species are necessary.

Table 4 summarizes the proposed biological activities based on bioinformatics tools and the experimental outcomes for the 12 peptides identified in the epidermal mucus of *S. lalandi* and *S. violacea*. Among them, peptide 5070 was the one having the most robust functional properties, encompassing antibacterial, antiparasitic, antioxidant, and respiratory burst increasing. It was followed by peptides 5069 (antibacterial, antiparasitic, and respiratory burst increasing) and 5065 (antibacterial, antiparasitic, and antioxidant), while other peptides did not exhibit the analyzed activities.

The examination of results from antimicrobial, antioxidant, and respiratory burst tests in leukocytes provides insights into amino acid composition patterns associated with the functional roles these molecules play in the mucus secretions of teleost fish. This analysis aids in understanding the specific contributions of these peptides to the innate immune system and overall health status of the fish species under study.

The observed correlation between antibacterial activity and the abundance of lysine (K), arginine (R), or glycine (G) residues in the peptide sequences aligns with previous research findings. In fact, studies on peptides, particularly those belonging to the cathelicidin family, have highlighted that an increased presence of these amino acids enhances antimicrobial efficacy. For instance, the CATH-BRALE peptide from the fish Brachymystax lenok, rich in R and G residues, was a potent inhibitor of the Gram-negative bacteria *Aeromonas salmonicida* and *Aeromonas hydrophila*, with a low MIC value of 9.38 μM *[20]*. Similarly, peptides such as As-CATH4 and As-CATH5 from the cathelicidin family have shown enhanced survival against antibiotic-resistant pathogens in the Chinese crab *Eriocheir sinensis [21].*

The presence of basic amino acids in the sequences of AMPs is closely linked to antibacterial mechanisms, primarily involving electrostatic interactions between these cationic residues and the anionic surface of the bacterial membranes *[22]*. This relationship has been established for fish AMPs, exemplified by peptides like omIL-8α and ssIL-8α. These peptides, rich in basic amino acids, were studied by Santana et al. *[23]*, demonstrating that their antibacterial action involves accumulation on bacterial surfaces followed by membrane permeabilization. While further investigation is required to unravel the exact mechanisms employed by the newly identified AMPs from the mucus of *S. lalandi* and *S. violacea*, the presence of basic amino acids in their composition suggests a potential involvement in membrane interactions as part of their antibacterial action. Therefore, the specific amino acid composition and secondary structure play a crucial role in determining the antimicrobial action of peptides.

The secondary structures, including alpha-helix or beta-sheet formations, are also important factors influencing how peptides interact with bacterial membranes or other targets, leading to their antimicrobial effects. Indeed, the presence of cysteine residues can contribute to antibacterial activity in certain peptides. Cysteine residues are unique among the amino acids because they contain a thiol group (-SH) that can form disulfide bonds with another cysteine residue. This capability to form disulfide bonds allows peptides to adopt specific structural conformations, such as stabilized beta-sheet structures *[24]*. For instance, a family of AMPs known as hepcidins, characterized by the presence of eight cysteine residues, includes peptides like HEP2p (GMKCKFCCNCCNLNGCGVCCRF) isolated from the flatfish turbot (*Scophthalmus maximus*). HEP2p has exhibited strong activity against Gram-negative bacteria such as *Edwardsiella tarda* (MIC = 1 µM) and *Vibrio anguillarum* (MIC =2 µM). Administration of HEP2p in turbot increased its survival against *V. anguillarum* infections *[25]*. In the present study, none of the peptides exhibiting antibacterial activity against the tested strains, contain disulfide bridges. Additionally, the only peptide containing a single cysteine residue in its sequence (5070: LGKFKGRSPC) exhibits a random coil structure, as determined by both in silico analysis and circular dichroism spectroscopy. Therefore, the presence of certain amino acids, along with the arrangement of them in the peptide sequence, contribute to their antimicrobial properties *[26,27]*. Moreover, the presence of cysteine residues may promote dimerization, potentially enhancing its antimicrobial potency, as evidenced in previous investigations. Nevertheless, more studies are necessary to evaluate the influence of dimer formation on the antibacterial efficacy of the 5070 peptide.

In this investigation, we employed *Caligus rogerresseyi* as a model to assess activity against parasites due to the absence of established methods for the *S. lalandi* or *S. violacea* species. The ability of certain peptides to exhibit antiparasitic activity against *C. rogercresseyi* is an interesting finding. The presence of basic amino acids, such as lysine and arginine, can contribute to the antiparasitic action of peptides, similar to their role in antibacterial activities. However, the obtained results regarding the presence of aromatic amino acids, such as tryptophan in peptide 5079, highlight the diversity of factors that can influence the functional properties of these peptides. Aromatic amino acids, like tryptophan and phenylalanine, can contribute to the hydrophobicity and structural stability of peptides. This aspect gains relevance in the context of antiparasitic activity, exemplified by fish AMPs like piscidin, which contain tryptophan and histidine in its sequence *[28,29]*. Interestingly, the peptide 5079 lacks antibacterial activity but still exhibits antiparasitic activity, which suggests that it might have multiple modes of action or specific interactions with the parasite that are distinct from its antibacterial mechanisms. Understanding the mechanism of action of peptides against ectoparasites is crucial for developing targeted and effective treatments. Unraveling how these peptides interact with and affect ectoparasites could provide valuable insights into their potential as therapeutic agents. Future research efforts should involve detailed studies on the molecular and cellular interactions between these peptides and ectoparasites. Overall, this study provides valuable insights into the multifunctional roles of these peptides in the antimicrobial defense mechanisms of fish, encompassing antibacterial, antiparasitic, and potentially other pathogen-killing activities.

Beyond their antimicrobial activity, AMPs can also act as signaling and chemotactic molecules, serving as a link between immune and adaptive responses *[30]*. When phagocytes encounter a pathogen, membrane perturbation and phagocytosis can occur, triggering the respiratory burst and subsequent cellular activation. As described by Zughaier et al. *[30]*, the formation of phagolysosomes and subsequent degranulation lead to the rapid release of these molecules into the phagocytic vacuole or the extracellular fluid. In such scenarios, AMPs can neutralize endotoxin-induced release of cellular cytokinins and nitric oxide. Therefore, inside phagocytes, AMPs enhance respiratory burst in leukocytes, promoting an increased release of reactive oxygen species (ROS). It is interesting to note that, in the present study, the peptides that stimulated respiratory burst, specifically peptides 5069, 5070, and 5079, were also the ones exhibiting potent antimicrobial activity. Similar findings have been reported for peptides derived from cathelicidins, such as HR-CATH identified in the tiger frog (*Hoplobatrachus rugulosus*). This peptide not only exhibited antibacterial activity against *Vibrio parahaemolyticus*, *Staphylococcus aureus*, and *Aeromonas hydrophila* but also induced increased chemotaxis and respiratory burst in RAW264.7 cells (a mouse leukemic monocyte/macrophage cell line) [31].

An additional example is the peptide NKHS27 derived from the seven-banded grouper (Hyporthodus septemfasciatus), which exhibited activity against both Gram-negative and Gram-positive bacteria. When applied in macrophage cultures, it not only demonstrated antimicrobial properties but also triggered respiratory burst in these cells. Moreover, NKHS27 positively influenced the expression of genes associated with the cellular immune response *[32]*. Similarly, the AMP LEAP-2A, identified in the liver of the cyprinid Hemibarbus labeo, demonstrated significant proinflammatory effects when combined with lipopolysaccharide (LPS) or phorbol 12-myristate 13-acetate (PMA). This combination induced a robust proinflammatory response in leukocytes, involving heightened activity of inducible nitric oxide synthase (iNOS), respiratory burst, and the proinflammatory cytokines IFN-γ, TNF-α, and IL-1β, as observed by Chen et al. *[33]*. This underscores that certain PAMs with antimicrobial activity can also modulate cellular responses in leukocytes, contributing to the elimination of pathogens, including the stimulation of respiratory burst, a mechanism observed in some peptides identified in the mucus of *S. lalandi* and *S. violacea* in the present study. This suggests a potential link between the ability to enhance the immune response (respiratory burst) and the peptides’ effectiveness in combating microbial threats. The co-ordination of these two activities may contribute to a robust defense mechanism against pathogens. Further research should explore the specific mechanisms underlying this correlation and shed light on the multifaceted roles of these peptides in the immune response. Up to this point, AMPs that have been characterized as “immunomodulatory” have not revealed a straightforward correlation between their amino acid composition and the regulatory function of proinflammatory leukocyte responses. Consequently, this remains an area that requires further investigation to unravel the precise mechanisms by which these peptides stimulate and modulate immune cells, such as cytokines up-regulation.

Antioxidants play a crucial role in mitigating oxidative stress by neutralizing harmful free radicals within the fish body, including mucosal tissues. In the realm of immune enhancement, these antioxidant molecules function to safeguard immune cells from oxidative damage, thereby promoting their optimal functionality. Peptides, known for their diverse biological activities, particularly those rich in aromatic amino acid residues such as tyrosine (Y), histidine (H), tryptophan (W), and phenylalanine (F), have been implicated in contributing to antioxidant properties *[34]*. Amino acids featuring aromatic rings in their side chains act as hydrogen donors to electron-deficient radicals, enhancing the capacity to eliminate free radicals. For instance, histidine and tryptophan residues donate hydrogen atoms, effectively eliminating radicals and forming stabilized indole or phenoxy radicals, thereby establishing a conjugated electron system *[35]*.

Peptides derived from marine fish, such as the peptides LHY and GAWA isolated from the Spanish sardine (*Sardinella aurita*), and those rich in phenylalanine and tryptophan obtained from whey protein hydrolysates have demonstrated antioxidant properties *[34]*. The present study aligns with this evidence, emphasizing the importance of aromatic amino acids for the antioxidant activity of peptides from the epidermal mucus of *S. lalandi* and *S. violacea*. Notably, the inclusion of cysteine in the sequences of antioxidant antimicrobial peptides further enhanced their efficacy. This is apparent in the case of peptides exhibiting high in vitro antioxidant capacity (5066, 5067, 5070, and 5077), as they not only contain aromatic amino acids but also feature a cysteine residue.

The antioxidant potential of cysteine residues in proteins and peptides stems from the sulfhydryl group in their side chain (–SH), possessing hydrogen-donating capacity against free radicals. Free radicals accept a hydrogen atom from the –SH group, transforming it into –S. This radical subsequently reacts with another -S or oxygen, converting into disulfide or alkylene sulfide (-SS-o SO_2_), thereby concluding the free radical chain reaction *[36]*. Hence, cysteine is regarded as essential for antioxidant activity due to its remarkable capability to neutralize free radicals. For instance, research conducted with peptides isolated from horse mackerel (*Magalaspis cordyla*) identified the ACFL peptide sequence, which exhibited potent DPPH activity *[36]*. Other fish-derived peptides, such as TCSP from Pacific cod (*Gadus macrocehalus*), have demonstrated the ability to eliminate intracellular reactive oxygen species (ROS), protect DNA from oxidative damage, and significantly enhance cell viability under oxidative stress *[37]*. Additionally, two peptides identified in *Decapterus maruadsi*, a fish from the Carangidae family, displayed robust antioxidant activity, attributed to the presence of cysteines in their composition, functioning similarly to glutathione *[38]*. Therefore, the antioxidant peptides identified in the mucus of *S. lalandi* and *S. violacea* play a crucial role in managing free radicals within the mucus membranes of these fish. 

## 4. Materials and Methods

### 4.1. Mucus Sampling from S. lalandi and S. violacea Juveniles

The mucus samples from juvenile *S. lalandi* and *S. violacea* were collected from 1-year-old individuals cultivated in the marine fish culture laboratory of the Universidad Católica del Norte. Initially, mucus was obtained from the fish cuticle by gently sliding and collecting it in a 50 mL tube, which was kept on ice throughout the process. The collected samples were then preserved at −80 °C until further use. 

The mucus samples were thawed on ice and then homogenized using Mini-Beadbeater-24 Biospec equipment (Houston, TX, USA). The mucus was homogenized in a lysis buffer (50 mM TRIS-HCl 10 mM EDTA, pH 8.0) containing a commercial protease inhibitor (Sigma-Aldrich, Tokyo, Japan). Subsequently, the homogenized mixture was centrifuged at 3000× *g* for 5 min at 4 °C. The resulting supernatant was further centrifuged at 12,000× *g* for 10 min at 4 °C and stored at −20 °C until analysis. 

For the isolation of peptide fractions, reverse-phase C-18 chromatographic columns (Themofisher, Tokyo, Japan) were utilized. The low-molecular-weight peptides (less than 10 kDa) were eluted with 10%, 20%, and 30% acetonitrile (ACN) in water *[39]*. Following elution, the samples were processed in SPEED-VAC equipment to remove organic solvents and subsequently lyophilized for further analysis.

### 4.2. Peptidomics Analysis

The peptide-enriched extracts from two mucus pools (100 µg lyophilized) for each fish species were subjected to analysis. The samples were suspended in 8 M urea in 50 mM NH_4_HCO_3_ and sonicated in an ultrasonic bath. Peptide quantification was carried out using the PierceTM Protein test. Subsequently, 10 µg of the peptide extracts underwent reduction (20 mM DTT in NH_4_HCO_3_ 50 mM; 60 min, 32 °C) and alkylation (55 mM iodoacetamide in NH_4_HCO_3_ 50 mM; 25 °C, 30 min, in the dark).

The resulting peptide mixtures were purified using a C18 tip (Polylcinc.) following the manufacturer’s protocol. Finally, the peptide solution was dried and stored at −20 °C for subsequent LC-MS/MS analysis. The elution gradient for peptides involved a progression from 1% to 40% acetonitrile (ACN) over 60 min, followed by an increase from 40% to 60% ACN in water by 10 min, with a flow rate of 250 mL/min. The masses and sequences of the peptides were determined using an LTQ-Orbitrap Velos mass spectrometer (Thermo Scientific, Markham, ON, Canada).

### 4.3. Bioinformatics Analysis

The peptide sequences were submitted to online databases utilizing predictive algorithms to theoretically categorize the biological activities of the peptides identified from fish mucus. The antimicrobial activity was assessed at https://aps.unmc.edu/preddiction/predict (accessed on 15 June 2023), while potential antioxidant properties were evaluated using http://lin.uestc.edu.cn/server/antioxipred (accessed on 15 June 2023). Moreover, putative secondary structure models of selected peptides were examined using the PEP-FOLD3 web server (http://bioserv.rpbs.univ-paris-diderot.fr/services/PEP-FOLD3, accessed on 16 June 2023) as outlined by Lamiable et al. [40]. Following the selection of the optimal model, the 3D structure of each peptide was constructed using PyMOL.

### 4.4. Solid-Phase Chemical Synthesis of Selected Peptides

In this study, 12 peptides with potential antimicrobial and antioxidant activities (refer to Table 1) were synthesized using a solid-phase synthesis strategy, employing rink-amide resin with a substitution degree of 0.6 meq/g. Throughout the coupling stages, Fmoc amino acids were attached using HBTU (2-(1H-Benzotriazole-1-yl)-1,1,3,3-tetramethylaminium hexafluorophosphate) and TBTU (2-(1H-Benzotriazole-1-yl)-1,1,3,3-tetramethylaminium tetrafluoroborate) as activators.

Following synthesis, the peptides were cleaved from the resin using a trifluoroacetic acid/triisopropylhydrosilane (TIS)/water mixture in a 95:2.5:2.5 ratio. The resulting peptides were precipitated with diethyl ether, reconstituted in Milli-Q water, and subjected to lyophilization. Purification was achieved using a C18 column and an acetonitrile gradient from 0 to 60% in water. 

The purity, exceeding 95%, and the molecular masses of the synthetic peptides were confirmed through reverse-phase high-performance liquid chromatography (RP-HPLC) and electrospray ionization mass spectrometry (ESI-MS), respectively.

The secondary structure analysis of the synthetic peptides was conducted via circular dichroism (CD) spectroscopy using a JASCO J-815 CD Spectrometer (Jasco Corp., Tokyo, Japan), following previously established procedures *[4]*. CD spectra of the peptides were acquired in both Milli-Q water and trifluoroethanol (TFE, 30% *v*/*v* in water) within the far ultraviolet (UV) range (190–250 nm). Quartz cuvettes with a path length of 0.1 cm and a bandwidth of 1 nm were utilized, with spectra recorded at a resolution of 0.1 nm. To obtain accurate measurements, the solvent contribution blank was subtracted from each sample spectrum. Molar ellipticity values were subsequently calculated for each peptide.

### 4.5. Antimicrobial Analysis

#### 4.5.1. Antibacterial Activity

Three bacterial species, namely *Escherichia coli* ML35, *Vibrio anguillarum* 507, and *Vibrio ordalii* DSM 19621, were utilized in this study. *E. coli* was inoculated in Trypticase Soy Agar (TSA) and incubated for 24 h at 37 °C and then transferred to Trypticase Soy Broth (TSB) and incubated for an additional 2 h at 37 °C under agitation. Meanwhile, *V. anguillarum* and *V. ordalii* cells were cultured in TSA supplemented with 1.5% NaCl and incubated at 25 °C for 24 h. Subsequently, they were subcultured in TSB-1.5% NaCl and incubated with gentle agitation for an additional 16 h at 25 °C. Optical density (OD) of the cultures at 600 nm was measured and dilutions were made to achieve a concentration of 1 × 10^7^ colony-forming units per milliliter (CFU/ mL).

Firstly, *E. coli* ML35 was utilized as a Gram-negative model to assess the antibacterial activity, following the methodology outlined by Santana et al. *[23]*. Briefly, cultures containing 1 × 10^7^ CFU/mL were exposed to synthetic peptides at a concentration of 100 µM and incubated for 1 h under previously described bacterial growth conditions. Subsequently, the treated cells were spread onto TSA plates and then incubated for 12 h at 37 °C (for *E. coli*) or 16 h at 37 °C. The resulting colonies were counted and bacterial survival was determined by calculating the CFU/mL. Additionally, untreated *E. coli* cultures and those treated with gentamicin at a concentration of 200 µM were used as controls. Independent experiments were repeated three times.

Following the antibacterial activity screening, peptides showing noticeable activity were selected to determine the minimum inhibitory concentration (MIC). Serial dilutions of these peptides were prepared, starting from a maximum concentration of 100 µM, and the same antibacterial activity test protocol was followed. The minimum inhibitory concentration (MIC) was determined following the protocol reported by Flóres-Castillo et al. *[41]*. For the microdilution method, 100 µL two-fold dilutions of synthetic peptides from 100 µM to 3.12 µM were added to 96-well polystyrene plates; 100 µL of bacteria inoculum (1 × 10^7^ CFU/well) was subsequently added and incubated in TSB (supplemented with 1.5% NaCl in the case of Vibrio strains) for 18h at the optimal growth temperature of bacteria strains. Finally, the MIC was determined by direct observation and by reading at 620 nm on a spectrophotometer. Strains were tested in duplicate with its respective controls: untreated bacteria (positive growth control), bacteria-free TSB medium (negative control), and gentamicin (200 µM).

#### 4.5.2. Antiparasitic Activity

As there is currently no established method for determining activity against parasites of the studied species, the ectoparasite copepod *Caligus rogerresseyi* was employed as a model. The antiparasitic activity was assessed following the protocol outlined by Montory et al. *[42]* at the CRC-Innovation Company in the city of Puerto Montt, Chile. For the in vitro determination, sensitivity bioassays were conducted using copepods grouped into a 120 μm mesh with a reduced volume of seawater. The parasite stock was distributed in 96-well-bottomed plates. Peptide effects were assessed at concentrations of 50 μm and 150 μm in seawater, with 200 μL inoculated into each of the 96 wells. Bioassays and plate maintenance were conducted at 12 °C and the effects were evaluated in eight repetitions. The larvae were exposed for up to 144 h at different peptide concentrations. This methodology aimed to determine the percentage of affected and/or dead individuals at each peptide concentration, as exposed by Montory et al. *[42]*.

### 4.6. Antioxidant Activity

To assess the antioxidant capacity of the synthetic peptides, two methods, namely 2,2-diphenyl-1-picrylhydrazyl (DPPH) for radical scavenging and total antioxidant capacity (TRAP), were employed.

#### 4.6.1. DPPH Method

The DPPH method followed the methodology reported by Madrid et al. *[43]*. A 50 μM DPPH solution was prepared in absolute ethanol, and peptides were prepared at 1 mg/mL in Milli-Q water (kept on ice). Glass cells for spectrometry were used, and 2.9 mL of the preprepared DPPH solution was added to each cell and the absorbance at 517 nm was recorded (T0). After this initial measurement, 100 μL of each peptide was added to the cells, and the mixture was incubated for 15 min, repeating this process three times. A cell containing only ethanol was used as the control. After 15 min, the cells’ absorbance at 517 nm was measured, and the results were used to calculate the percentage of radical scavenging capacity (SRC%):

SRS% = [(T0 − sample absorbance)/T0] × 100.

#### 4.6.2. TRAP Method

The total antioxidant capacity was determined using the TRAP method, following the procedure described by Leyton et al. *[44]*. A 10 mM 2,2’azobis (2-amidopropane) dihydrochloride (AAPH) solution was prepared and mixed with 150 µM 2,2′-azino-bis(3-ethylbenzothiazoline-6-sulfonic acid) (ABTS) solution in 100 mM phosphate buffer saline (pH 7.4). The mixture was incubated at 45 °C for 30 min to generate the ABTS radical. Then, 10 µL of peptides (1 mg/mL) were mixed with 990 µL of the ABTS radical solution, and absorbance was measured kinetically over 50 s at 734 nm. The percentage of inhibition of the radical (IR) was calculated using the equation: IR = [(A_0_ − A_50_)/A_0_] × 100, where A_0_ is the absorbance at 0 s and A_50_ is the absorbance at 50 s. The IR percentages were then extrapolated onto a Trolox® curve and the results were expressed in millimolar (mM) of antioxidant capacity equivalents of Trolox® (TEAC mM). These TEAC results were compared to the antioxidant activity of 4340 peptide from marine origin *[19].*

### 4.7. Extraction of Head Kidney Leukocytes and Respiratory Burst Analysis

Two fish of 2 kg of total body mass (*S. lalandi*) at the Fish Laboratory of the Universidad Católica del Norte were chosen for the extraction of blood via flow puncture, followed by the extraction of the head kidney. The tissue was kept cold in L-15 medium supplemented with 2% serum and antibiotics (1% commercial solution of 100 U/mL of penicillin, 100 mg/mL of streptomycin, and 5 µg/mL of gentamycin). The extraction of macrophages was performed using the method described by Stolen et al. *[45]*, with some modifications.

The kidneys were manually crushed using sterile and filtered plastic micropistols through a 100 µm nylon membrane with L-15 medium supplemented with serum and antibiotics. After centrifuging at 450× *g* for 10 min at 4 °C, the supernatant was removed and the cells were suspended in the medium and subjected to a Ficoll gradient (Lymphoprep; 1:1). Subsequently, it was centrifuged at 600× *g* without acceleration for 30 min at 4 °C and the interface containing the macrophages was carefully transferred with a sterile Pasteur pipette to a new glass tube. The cells were then diluted in 1 mL of L-15 medium without supplement and centrifuged (450× *g*, 10 min, 4 °C) to wash out the residual Ficoll. After resting for 10 min, the sample was centrifuged at 685× *g* for 10 min at 4 °C. The supernatant was removed, and another washing step was performed and then centrifugated. The cells were resuspended again in 1 mL of L-15 medium. Subsequently, viable cells were identified by vital Trypan Blue staining (10 µL cells and 40 µL staining) in a Neubauer’s chamber. Finally, the concentration was adjusted to 1 × 10^6^ cells/mL and the cultures were prepared in 96-well-bottomed plates with 1 × 10^5^ macrophages per well, using L-15 medium without serum but with antibiotics to promote cell adhesion to the polystyrene plates. After a 2-h incubation at 22 °C, the unattached cells were removed by two washings with culture medium. The macrophage cultures were then incubated overnight at 22 °C with L-15 medium supplemented with serum and antibiotics.

The production of intracellular superoxides by phagocytes was assessed through the reduction of tetrazolium nitroblue tetrazolium (NBT) reagent (Sigma), following the protocols outlined by Stolen et al. *[45,46]* and Boesen et al. *[46]*. Initially, macrophage cultures in the 96-well plate were washed with nonsupplemented L-15 medium and Hanks’ balanced salt solution (HBSS) to eliminate any residual antibiotic. Subsequently, 100 µL of NBT dissolved to a concentration of 1 mg/mL in HBS was added to each well. In the initial assessment, 1 µg/µL of the peptide extract was used but doses were subsequently adjusted based on prior studies *[19]*. The cultures were then incubated at 22 °C for 45 min. As a positive control, wells were inoculated with 1 µg/mL of phorbol myristate acetate (PMA; Sigma). To assess the specificity of the reaction, 300 µL of superoxide dismutase (SOD; Sigma) was added to one of the wells treated with PMA. After the incubation period, the supernatant was removed and 70% methanol in water was added. Methanol was allowed to evaporate under an extraction hood overnight. The phagocyte form was solubilized by adding 120 µL of 2 M KOH and 140 µL of dimethyl sulfoxide (DMSO; Sigma). Absorbance at 620 nm was then measured using an EPOCH spectrophotometer. Finally, the results were expressed as a stimulation index, obtained by dividing the value of each treatment by the control without stimulation.

### 4.8. Statistical Analysis

Statistical analysis was performed in R v4.3.1 The normal distribution of all data was assessed by Shapiro–Wilks test. Statistical significance of differences in antibacterial activity was determined with a one-way ANOVA test. For antiparasitic and respiratory burst activities, two-way ANOVA tests were employed, considering significance at *p* = 0.05.

For antioxidant activity, a Chi-squared test was carried out. For the TRAP and DPPH methods, a Kruskal–Wallis test was used for multiple independent samples with a significance level of *p* = 0.01.

## 5. Conclusions

The present study successfully identified and demonstrated the biological activity of nine peptides within the mucus secretions of marine fish native to Chile. Specifically, five of them were found in the mucus of juveniles of *S. violacea*, while the remaining four were identified in the mucus of *S. lalandi*.

All the characterized peptides exhibited a disordered or random coil secondary structure, indicating that their biological activity was closely related to their amino acid composition. Notably, those peptides displaying strong antimicrobial activity showcased a higher abundance of basic amino acids, like lysine and arginine, coupled with hydrophobic residues, such as phenylalanine and tryptophan. This amphipathic nature likely contributes to their efficacy in combating pathogens. Moreover, these peptides demonstrated the additional capability of stimulating respiratory burst in fish leukocytes.

Furthermore, the obtained results highlight the significance of aromatic amino acids, along with cysteine, in significantly enhancing the antioxidant activity of the peptides present in mucosal secretions. This dual role of certain peptides in antimicrobial defense and antioxidant protection underscores their multifunctional nature.

While the present study has provided valuable insights, it is imperative to conduct further investigations to assess additional biological activities of these peptides, such as their potential antifungal and antiviral functions. This research is ongoing, being essential for expanding the understanding of the peptides’ broader roles and their relevance in determining the health status of cultured fish.

## Figures and Tables

**Table 1:** Characterization of chemically synthesized peptides identified from fish epidermal mucus.

Peptide ID	Residue	Fish Specie	MW * (Da)	Sequence	RT * (min)
5065	8	*Seriolella violacea*	977.29	FGVKWVKN	5.5
5066	9	*Seriolella violacea*	905.11	CTAGETAPR	4.0
5067	9	*Seriolella violacea*	1038.31	CTKGETFPR	5.0
5068	10	*Seriola lalandi*	1087.35	SRSALSLRTP	5.5
5069	10	*Seriola lalandi*	1186.49	SRSALWLRTP	5.8
5070	10	*Seriola lalandi*	1092.46	LGKFKGRSPC	5.8
5076	9	*Seriola lalandi*	974.28	LGLFKGRSP	5.5
5077	9	*Seriola lalandi*	1064.38	AERLTPCFK	5.6
5078	8	*Seriola lalandi*	904.12	AERLTPAF	5.8
5079	9	*Seriolella violacea*	1042.32	KWTGNLAPR	5.4
5080	9	*Seriolella violacea*	943.18	KSTGNLAPR	4.3
5081	8	*Seriolella violacea*	890.21	FGVKVVKN	5.0

* MW: molecular weight; RT: retention time.

**Table 2:** Minimum inhibitory concentration (MIC) of selected peptides against bacterial strains.

Peptide	MIC (µM)		
	*Vibrio anguillarum*	*Vibrio ordalii*	*Escherichia coli*
5065	12.5	6.25	25
5069	12.5	12.5	25
5070	25	25	25
5076	>100	25	25

**Table 3:** Evaluation of antioxidant activity in synthetic peptides by DPPH and TRAP methods.

Peptide ID	DPPH (%RSC)				TRAP (TEAC µM)		
5065	6.883	±	1.204		7.078	±	0.914
5066	13.693	±	0.848	*	15.845	±	0.043
5067	31.031	±	1.233	***	53.164	±	3.251 **
5068	3.480	±	0.315		N.D.		
5069	4.564	±	0.695		6.428	±	0.998
5070	11.202	±	0.388	*	53.249	±	4.597 **
5076	2.048	±	0.102		0.813	±	0.358
5077	16.509	±	0.723	**	91.621	±	7.722 ***
5078	3.416	±	0.367		3.376	±	0.156
5079	3.526	±	1.060		5.544	±	0.355
5080	2.454	±	0.704		2.137	±	0.094
5081	5.084	±	1.051		2.287	±	0.536
4340	5.479	±	0.630		10.020	±	0.010

%RSC = radical scavenging capacity percentage values; TEAC = equivalent antioxidant capacity of TROLOX; N.D. = not determined. 4340 = reference antioxidant peptide (GPEPTGPTGAPQWLR). * *p*  <  0.05; ** *p*  <  0.01; *** *p*  <  0.001.

**Table 4:** Summary and comparative analysis of simulated and experimentally determined biological activities for synthesized peptides.

Peptide ID	Fish Specie	Sequence	Theoretical Activity	Proven Biological Activity
5065	*Seriolella violacea*	FGVKWVKN	AM	AB/AOX/AP
5066	*Seriolella violacea*	CTAGETAPR	AM/AOX	AOX
5067	*Seriolella violacea*	CTKGETFPR	AM/AOX	AOX/IM
5068	*Seriola lalandi*	SRSALSLRTP	AM	N.D.
5069	*Seriola lalandi*	SRSALWLRTP	AM	AB/AP/IM
5070	*Seriola lalandi*	LGKFKGRSPC	AM	AB/AOX/AP/IM
5076	*Seriola lalandi*	LGLFKGRSP	AM	AB/IM
5077	*Seriola lalandi*	AERLTPCFK	AM	AOX
5078	*Seriola lalandi*	AERLTPAF	AM/AOX	N.D.
5079	*Seriolella violacea*	KWTGNLAPR	AM/AOX	AP
5080	*Seriolella violacea*	KSTGNLAPR	AM/AOX	N.D.
5081	*Seriolella violacea*	FGVKVVKN	AM/AOX	AOX

AM: antimicrobial activity; AOX: antioxidant activity; B: antibacterial activity; AP: antiparasitic activity; IM: immunomodulatory activity; N.D. = not determined.

## Data Availability

The original data presented in the study are included in the article/Supplementary Material; further inquiries can be directed to the corresponding author.

## Supplementary Materials

The following supporting information can be downloaded at: https://www.mdpi.com/article/10.3390/md22060248/s1, Figure S1: RP-HPLC chromatogram of synthetic peptides; Figure S2: ESI MS/MS spectrum of the [M]^+^ molecular ion of synthetic peptides; Figure S3: 3D-structure model of peptides.

## References

[1] Nerici C., Merino G., Silva A. (2012). Effects of Two Temperatures on the Oxygen Consumption Rates of *Seriolella violacea* (Palm Fish) Juveniles under Rearing Conditions. Aquac. Eng..

[2] Alveal K., Silva A., Lohrmann K.B., Viana M.T. (2019). Morphofunctional Characterization of the Digestive System in the Palm Ruff Larvae, *Seriolella violacea* under Culture Conditions. Aquaculture.

[3] Allen P.J., Brokordt K., Oliva M., Alveal K., Flores H., Álvarez C.A. (2021). Physiological Insights for Aquaculture Diversification: Swimming Capacity and Efficiency, and Metabolic Scope for Activity in Cojinoba *Seriolella violacea*. Aquaculture.

[4] Álvarez C.A., Alvarado J.F., Farías M., Cárcamo C.B., Flores H., Guzmán F., Martín S.S., Varas J., Messina S., Acosta F. (2023). First Insights about Orexigenic Activity and Gastrointestinal Tissue Localization of Ghrelin from Corvina Drum (*Cilus gilberti*). Aquaculture.

[5] Miccoli A., Saraceni P.R., Scapigliati G. (2019). Vaccines and Immune Protection of Principal Mediterranean Marine Fish Species. Fish Shellfish Immunol..

[6] Sicuro B., Luzzana U. (2016). The State of Seriola Spp. Other Than Yellowtail (*S. quinqueradiata*) Farming in the World. Rev. Fish. Sci. Aquac..

[7] Ji Q., Wang S., Ma J., Liu Q. (2020). A Review: Progress in the Development of Fish Vibrio Spp. Vaccines. Immunol. Lett..

[8] Miranda C.D., Rojas R. (1996). Vibriosis in the Flouder *Paralichithys adspersus* (Steindachner, 1867) in Captivity. Rev. Biol. Mar..

[9] Rubio-Godoy M. (2010). Inmunología de Los Peces Óseos: Revisión Teleost Fish Immunology: Review. Rev. Mex. Cienc. Pecu..

[10] Concha K., Olivares P., Fonseca-Salamanca F., Sanchez R., Serrano F., Parodi J. (2017). Mucogenic Additives for the Control of Caligus Rogercresseyi in Atlantic Salmon (*Salmo salar*). Rev. Investig. Vet. Peru.

[11] Benhamed S., Guardiola F.A., Mars M., Esteban M.Á. (2014). Pathogen Bacteria Adhesion to Skin Mucus of Fishes. Vet. Microbiol..

[12] Aranishi F., Nakane M. (1997). Epidermal proteases of the Japanese eel. Fish Physiol. Biochem..

[13] Sanahuja I., Fernández-Alacid L., Ordóñez-Grande B., Sánchez-Nuño S., Ramos A., Araujo R.M., Ibarz A. (2019). Comparison of Several Non-Specific Skin Mucus Immune Defences in Three Piscine Species of Aquaculture Interest. Fish Shellfish Immunol..

[14] Zhang L., Falla T.J. (2009). Cosmeceuticals and Peptides. Clin. Dermatol..

[15] Shabir U., Ali S., Magray A.R., Ganai B.A., Firdous P., Hassan T., Nazir R. (2018). Fish Antimicrobial Peptides (AMP’s) as Essential and Promising Molecular Therapeutic Agents: A Review. Microb. Pathog..

[16] Valero Y., Saraiva-Fraga M., Costas B., Guardiola F.A. (2020). Antimicrobial Peptides from Fish: Beyond the Fight against Pathogens. Rev. Aquac..

[17] Reddy K.V.R., Yedery R.D., Aranha C. (2004). Antimicrobial Peptides: Premises and Promises. Int. J. Antimicrob. Agents.

[18] Medina M., Prado-Barragán B., Martínez-Hernández A., Ruíz H.A.A., Rodríguez R.M., Contreras-Esquivel A., Aguilar C.N. (2019). Péptidos Bio-Funcionales: Bioactividad, Producción y Aplicaciones. Bio-Functional Peptides: Bioactivity, Production and Applications. Rev. Científica Univ. Autónoma Coahuila.

[19] Zhou X., Wang C., Jiang A. (2012). Antioxidant Peptides Isolated from Sea Cucumber *Stichopus japonicus*. Eur. Food Res. Technol..

[20] Li Z., Zhang S., Gao J., Guang H., Tian Y., Zhao Z., Wang Y., Yu H. (2013). Structural and Functional Characterization of CATH_BRALE, the Defense Molecule in the Ancient Salmonoid, *Brachymystax lenok*. Fish Shellfish Immunol..

[21] Guo Z., Qiao X., Cheng R., Shi N., Wang A., Feng T., Chen Y., Zhang F., Yu H., Wang Y. (2017). As-CATH4 and 5, Two Vertebrate-Derived Natural Host Defense Peptides, Enhance the Immuno-Resistance Efficiency against Bacterial Infections in Chinese Mitten Crab, *Eriocheir sinensis*. Fish Shellfish Immunol..

[22] Carvajal-Rondanelli P., Aróstica M., Marshall S.H., Albericio F., Álvarez C.A., Ojeda C., Aguilar L.F., Guzmán F. (2016). Inhibitory Effect of Short Cationic Homopeptides against Gram-Negative Bacteria. Amino Acids.

[23] Santana P.A., Salinas N., Álvarez C.A., Mercado L.A., Guzmán F. (2018). Alpha-Helical Domain from IL-8 of Salmonids: Mechanism of Action and Identification of a Novel Antimicrobial Function. Biochem. Biophys. Res. Commun..

[24] Álvarez C.A., Guzmán F., Cárdenas C., Marshall S.H., Mercado L. (2014). Antimicrobial Activity of Trout Hepcidin. Fish Shellfish Immunol..

[25] Zhang J., Yu L.p., Li M.f., Sun L. (2014). Turbot (*Scophthalmus maximus*) Hepcidin-1 and Hepcidin-2 Possess Antimicrobial Activity and Promote Resistance against Bacterial and Viral Infection. Fish Shellfish Immunol..

[26] Lorenzon E.N., Piccoli J.P., Santos-Filho N.A., Cilli E.M. (2019). Dimerization of Antimicrobial Peptides: A Promising Strategy to Enhance Antimicrobial Peptide Activity. Protein Pept. Lett..

[27] Ohno M.K., Kirikae T., Yoshihara E., Kirikae F., Ishida I. (2020). Addition of L-Cysteine to the N- or C-Terminus of the All-D-Enantiomer [D(KLAKLAK)2] Increases Antimicrobial Activities against Multidrug-Resistant *Pseudomonas aeruginosa*, *Acinetobacter baumannii* and *Escherichia coli*. PeerJ.

[28] Fernandes J.M.O., Ruangsri J., Kiron V. (2010). Atlantic Cod Piscidin and Its Diversification through Positive Selection. PLoS ONE.

[29] Colorni A., Ullal A., Heinisch G., Noga E.J. (2008). Activity of the Antimicrobial Polypeptide Piscidin 2 against Fish Ectoparasites. J. Fish. Dis..

[30] Zughaier S.M., Shafer W.M., Stephens D.S. (2005). Antimicrobial Peptides and Endotoxin Inhibit Cytokine and Nitric Oxide Release but Amplify Respiratory Burst Response in Human and Murine Macrophages. Cell Microbiol..

[31] Chen J., Lin Y.F., Chen J.H., Chen X., Lin Z.H. (2021). Molecular Characterization of Cathelicidin in Tiger Frog (*Hoplobatrachus rugulosus*): Antimicrobial Activity and Immunomodulatory Activity. Comp. Biochem. Physiol. Part—C Toxicol. Pharmacol..

[32] Wang C.b., Yan X., Wang G.h., Liu W.q., Wang Y., Hao D.f., Liu H.m., Zhang M. (2023). NKHs27, a Sevenband Grouper NK-Lysin Peptide That Possesses Immunoregulatory and Antimicrobial Activity. Fish Shellfish Immunol..

[33] Chen J., Lv Y.P., Dai Q.M., Hu Z.H., Liu Z.M., Li J.H. (2019). Host Defense Peptide LEAP-2 Contributes to Monocyte/Macrophage Polarization in Barbel Steed (*Hemibarbus labeo*). Fish Shellfish Immunol..

[34] Jiang B., Zhang X., Yuan Y., Qu Y., Feng Z. (2017). Separation of Antioxidant Peptides from Pepsin Hydrolysate of Whey Protein Isolate by ATPS of EOPO Co-Polymer (UCON)/Phosphate. Sci. Rep..

[35] Kalyanaraman B. (2013). Teaching the Basics of Redox Biology to Medical and Graduate Students: Oxidants, Antioxidants and Disease Mechanisms. Redox Biol..

[36] Sampath Kumar N.S., Nazeer R.A., Jaiganesh R. (2012). Purification and Identification of Antioxidant Peptides from the Skin Protein Hydrolysate of Two Marine Fishes, Horse Mackerel (*Magalaspis cordyla*) and Croaker (*Otolithes ruber*). Amino Acids.

[37] Ngo D.H., Ryu B.M., Vo T.S., Himaya S.W.A., Wijesekara I., Kim S.K. (2011). Free Radical Scavenging and Angiotensin-I Converting Enzyme Inhibitory Peptides from Pacific Cod (*Gadus macrocephalus*) Skin Gelatin. Int. J. Biol. Macromol..

[38] Jiang H., Tong T., Sun J., Xu Y., Zhao Z., Liao D. (2014). Purification and Characterization of Antioxidative Peptides from Round Scad (*Decapterus maruadsi*) Muscle Protein Hydrolysate. Food Chem..

[39] Guzmán F., Gauna A., Roman T., Luna O., Álvarez C., Pareja-Barrueto C., Mercado L., Albericio F., Cárdenas C. (2021). Tea Bags for Fmoc Solid-Phase Peptide Synthesis: An Example of Circular Economy. Molecules.

[40] Lamiable A., Thévenet P., Rey J., Vavrusa M., Derreumaux P., Tufféry P. (2016). PEP-FOLD3: Faster de Novo Structure Prediction for Linear Peptides in Solution and in Complex. Nucleic Acids Res..

[41] Flórez-Castillo J.M., Rondón-Villareal P., Ropero-Vega J.L., Mendoza-Espinel S.Y., Moreno-Amézquita J.A., Méndez-Jaimes K.D., Farfán-García A.E., Gómez-Rangel S.Y., Gómez-Duarte O.G. (2020). Ib-M6 Antimicrobial Peptide: Antibacterial Activity against Clinical Isolates of Escherichia Coli and Molecular Docking. Antibiotics.

[42] Montory J.A., Chaparro O.R., Averbuj A., Salas-Yanquin L.P., Büchner-Miranda J.A., Gebauer P., Cumillaf J.P., Cruces E. (2020). The Filter-Feeding Bivalve *Mytilus chilensis* Capture Pelagic Stages of *Caligus rogercresseyi*: A Potential Controller of the Sea Lice Fish Parasites. J. Fish. Dis..

[43] Madrid A.M., Espinoza L.J., Mellado M.A., Osorio M.E., Montenegro I.J., Jara C.E. (2012). Evaluation of the Antioxidant Capacity of *Psoralea glandulosa* L. (*Fabaceae*) EXTRACTS. J. Chil. Chem. Soc..

[44] Leyton M., Mellado M., Jara C., Montenegro I., González S., Madrid A. (2015). Free Radical-Scavenging Activity of Sequential Leaf Extracts of *Embothrium coccineum*. Open Life Sci..

[45] Stolen J.S., Fletcher T.C., Anderson D.P., Roberson B.S., van Muiswinkel W.B. (1990). Techniques in Fish Immunology.

[46] Boesen H.T., Larsen M.H., Larsen J.L., Ellis A.E. (2001). In Vitro Interactions between Rainbow Trout (*Oncorhynchus mykiss*) Macrophages and Vibrio *Anguillarum serogroup* O2a. Fish Shellfish Immunol..

