# Supplementary material for: Evaluation of the Biological Activities of Peptides from Epidermal Mucus of Marine Fish Species from Chilean Aquaculture
Source: Mar Drugs. 2024 May 28;22(6):248. doi: 10.3390/md22060248 (PMC11204461; doi:10.3390/md22060248)
Supplement: Supplementary file 1 [file marinedrugs-22-00248-s001.zip › marinedrugs-3024269-supplementary.pdf]

marine drugs

Type of the Paper (Article)

Evaluation of the biological activities of peptides from epidermal mucus of marine fish species from Chilean aquaculture

Claudio A. Álvarez[1,2¥], Teresa Toro-Araneda[2¥], Juan Pablo Cumillaf[3], Belinda Vega[2], María José Tapia[2], Tanya Roman[4], Constanza Cárdenas[4], Valentina Córdova-Alarcón[1,8], Carlos Jara-Gutiérrez[5,6], Paula A. Santana[7*] and Fanny Guzmán[4*]

[1] Laboratorio de Cultivo de Peces Marinos, Facultad de Ciencias del Mar, Universidad Católica del Norte, Coquimbo 1781421, Chile; claudio.alvarez@ucn.cl
[,2] Laboratorio de Fisiología y Genética Marina (FIGEMA), Centro de Estudios Avanzados en Zonas Áridas (CEAZA), Coquimbo 1781421, Chile; teresa.toro@alumnos.ucn.cl (T.T.-A.); belinda.vega@ceaza.cl (B.V.); maria.tapia01@alumnos.ucn.cl (M.J.T.); valentina.cordova@ug.uchile.cl (V.C.-A.)
[3] CRC Innovación, Puerto Montt 5507642, Chile; jpnenen@gmail.com
[4] Núcleo Biotecnología Curauma, Pontificia Universidad Católica de Valparaíso, Valparaíso 2373223, Chile; tanya.roman.b@mail.pucv.cl (T.R.); constanza.cardenas@pucv.cl (C.C.); fanny.guzman@pucv.cl
[5] Genomics on the Wave SpA, Viña del Mar 2520056, Chile; valentina.cordova@ug.uchile.cl
[6] Centro Interdisciplinario de Investigación Biomédica e Ingeniería para la Salud—MEDING, Universidad de Valparaíso, Valparaíso 2362905, Chile; carlos.jara@uv.cl
[7] Facultad de Medicina, Escuela de Kinesiología, Universidad de Valparaíso, Valparaíso 2362905, Chile
[8] Instituto de Ciencias Aplicadas, Facultad de Ingeniería, Universidad Autónoma de Chile, Santiago 8910060, Chile
† The authors contributed equally to this work.
* Correspondence: paula.santana@uautonoma.cl (P.A.S.); fanny.guzman@pucv.cl (F.G.)

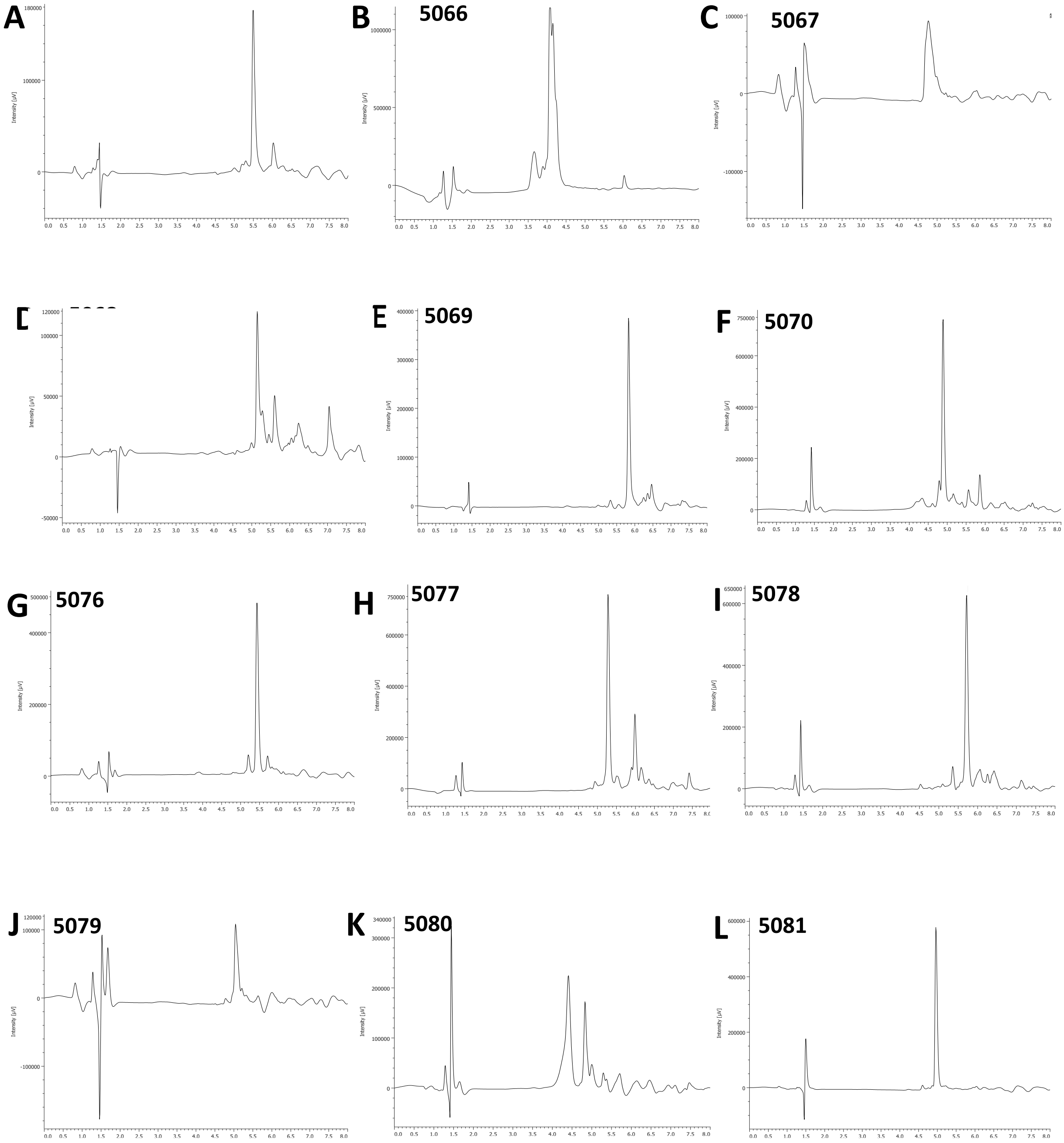

Figure S1. RP-HPLC chromatogram of synthetic peptides.

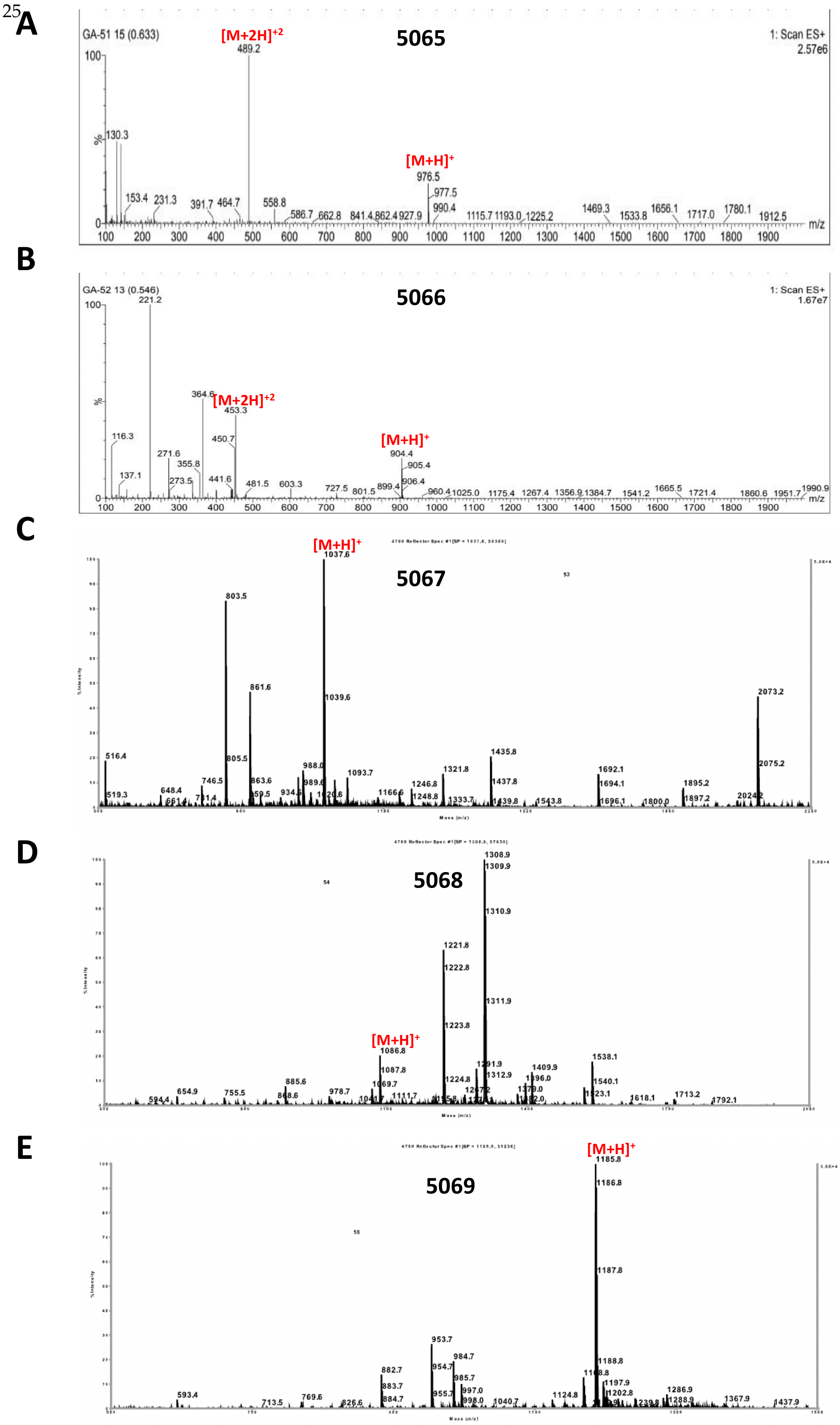
A
GA-51 15 (0.633)
[M+2H]+2
489.2
5065
1: Scan ES+
2.57e6
[M+H]+
976.5
m/z
B
GA-52 13 (0.546)
221.2
5066
1: Scan ES+
1.67e7
[M+2H]+2
453.3
[M+H]+
904.4
m/z
C
[M+H]+
1037.6
5067
D
5068
[M+H]+
1086.8
E
[M+H]+
1185.8
5069

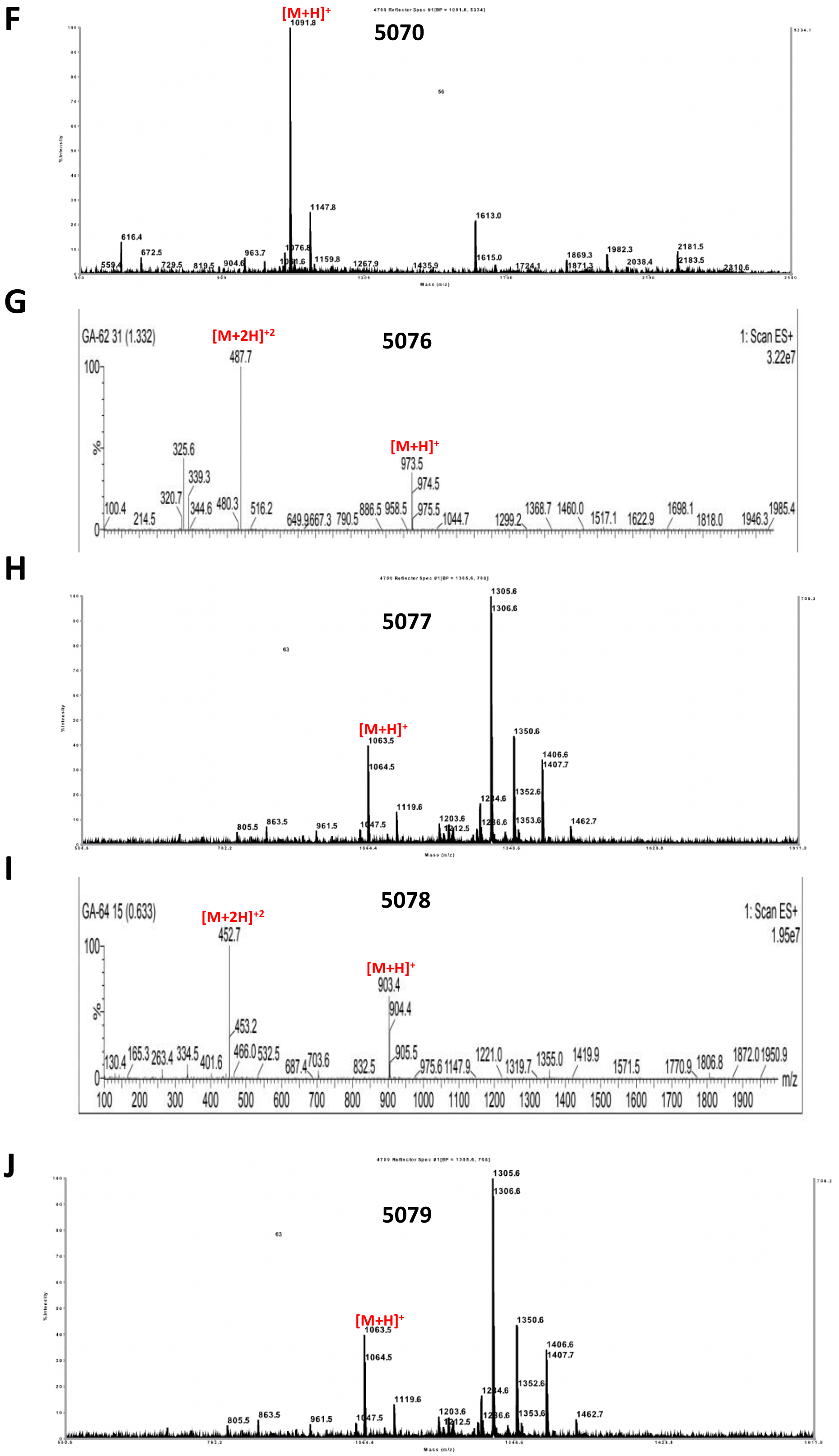

Figure S2. ESI MS/MS spectrum of the [M]$^+$ molecular ion of synthetic peptides.

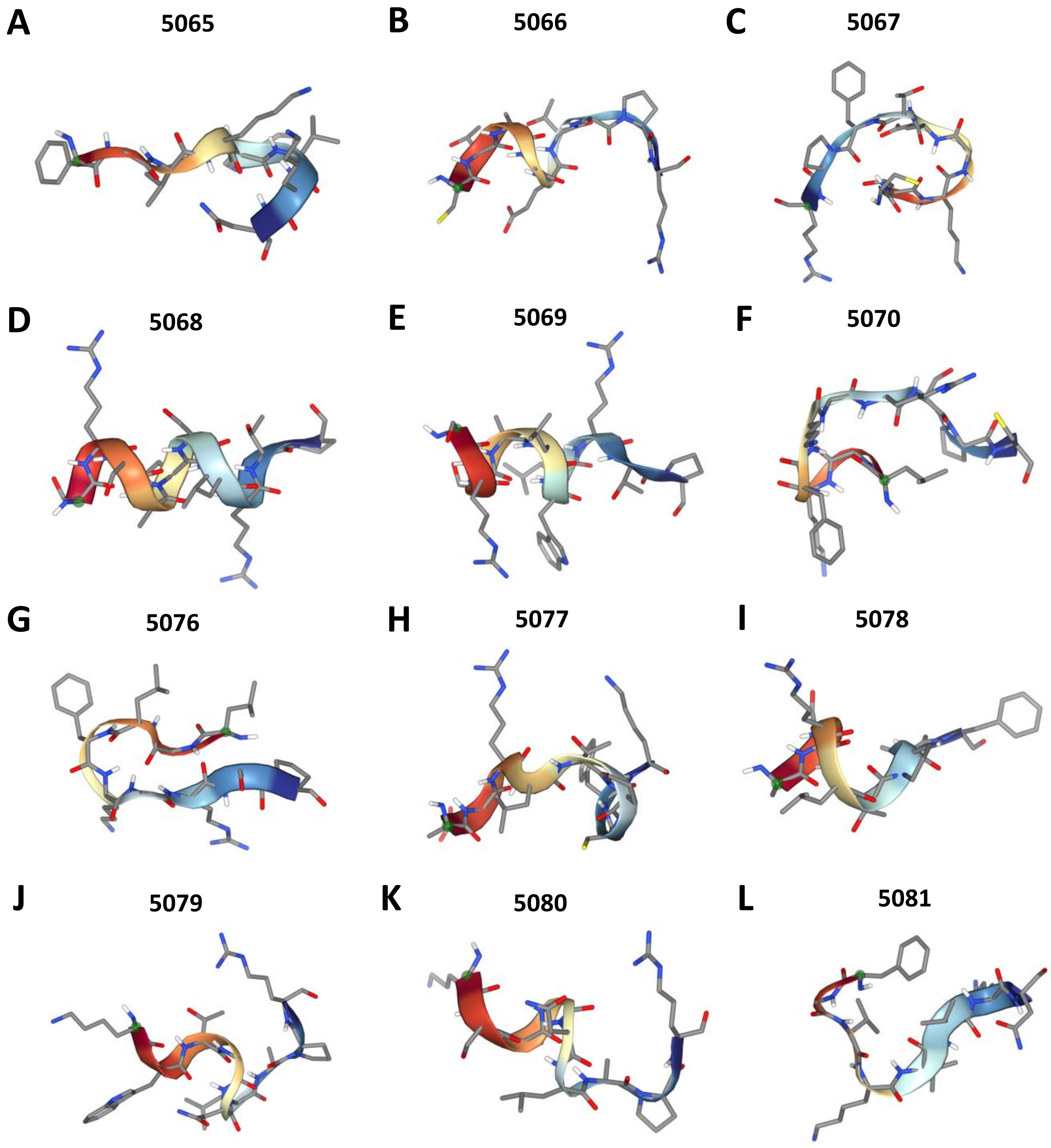

Figure S3. 3D-structure model of peptides.
